# Modulation of plant-mediated interactions between herbivores of different feeding guilds: Effects of parasitism and belowground interactions

**DOI:** 10.1038/s41598-018-32131-9

**Published:** 2018-09-26

**Authors:** Teresa Vaello, Sandeep J. Sarde, Mª Ángeles Marcos-García, Jetske G. de Boer, Ana Pineda

**Affiliations:** 1 Unidad Asociada IPAB (UA-CSIC), Instituto Universitario de Investigación CIBIO, University of Alicante. Ctra. San Vicente del Raspeig s/n, E-03690 Alicante, Spain; 2 Laboratory of Entomology, University of Wageningen, P.O. Box 16, 6700 AA Wageningen, The Netherlands; 3 Department of Terrestrial Ecology, Netherlands Institute of Ecology (NIOO-KNAW), P.O. Box 50, 6700 AB Wageningen, The Netherlands

## Abstract

Herbivory affects subsequent herbivores, mainly regulated by the phytohormones jasmonic (JA) and salicylic acid (SA). Additionally, organisms such as soil microbes belowground or parasitoids that develop inside their herbivorous hosts aboveground, can change plant responses to herbivory. However, it is not yet well known how organisms of trophic levels other than herbivores, below- and above-ground, alter the interactions between insect species sharing a host plant. Here, we investigated whether the parasitoid *Aphidius colemani* and different soil microbial communities (created through plant-soil feedbacks) affect the JA and SA signalling pathways in response to the aphid *Myzus persicae* and the thrips *Frankliniella occidentalis*, as well as subsequent thrips performance. Our results show that the expression of the JA-responsive gene Ca*PINII* in sweet pepper was more suppressed by aphids than by parasitised aphids. However, parasitism did not affect the expression of Ca*PAL1*, a biosynthetic gene of SA. Furthermore, aphid feeding enhanced thrips performance compared with uninfested plants, but this was not observed when aphids were parasitised. Soils where different plant species were previously grown, did not affect plant responses or the interaction between herbivores. Our study shows that members of the third trophic level can modify herbivore interactions by altering plant physiology.

## Introduction

Plants have evolved sophisticated strategies to defend themselves against pathogens and herbivorous insects. Plants regulate defence signalling pathways mainly through the production of the phytohormones jasmonic acid (JA) and salicylic acid (SA). The JA-signalling pathway is mainly induced by necrotrophic pathogens and insects that inflict cellular damage such as chewing or cell- content feeding herbivores, whereas the SA-signalling pathway is induced by biotrophic pathogens and phloem-feeding insects^1–3^. In recent years, new advances have shown how the plant response to one attacker can influence the performance of herbivores sharing the same host plant, a phenomenon that is driven by positive or negative interactions between these two signalling pathways^4–7^. For example, previous herbivory from phloem feeders that induce a SA response, often facilitates the later performance of herbivores that induce a JA response in the plant, via negative cross-talk of these signalling pathways^4–8^. However, it remains unknown how organisms of trophic levels other than herbivores interacting with the same plant will affect these often-facilitative interactions between herbivorous insects from different feeding guilds inducing different signalling pathways.

Aboveground, herbivorous insects can be attacked by parasitoids whose larvae develop inside their hosts. It is well established that plants can influence parasitoid performance^9,10^, but it has only recently been demonstrated that parasitoids can also affect plant responses to herbivory. Plants that are attacked by parasitised caterpillars show altered herbivore-induced plant responses, such as the emission of volatiles or the expression of certain defence genes^11–14^. Even more interesting is that the altered plant responses due to caterpillar parasitism can also affect other insects, such as hyperparasitoids^13^, moths species^11^, and parasitoids developing in another herbivore^15^. At present, our knowledge on how the third trophic level affects interactions between multiple plant attackers via the plant’s response is still limited to brassicaceous plants and associated caterpillars, thus it remains unknown whether these findings can be extended to other groups of plants and herbivores, especially those that induce different plant signalling pathways (such as phloem feeders). Thus, the posssibility of aphid parasitoids as modulators of plant defences, or how these changes may influence other feeding guilds, such as cell-feeding insects, is still unknown.

Belowground, plants interact with soil microbes, such as mycorrhizal fungi and plant growth promoting rhizobacteria, that can enhance plant growth and induce systemic resistance against different attackers^16–19^. There is increased awareness that interactions with the full community of soil microbes, the so-called soil microbiome, contribute to a plant’s extended phenotype, and can thereby affect herbivorous insects. For example, soil microbial communities can influence primary and secondary plant metabolite concentration^20–22^, affecting above-ground insects^23–26^. In turn, plants can also shape the biotic (e.g. microbes) and abiotic characteristics of the soil they grow in, and these changes can affect the performance of plants that grow in that soil subsequently. This phenomenon has been termed “plant-soil feedbacks” (PSF)^27–29^. The concept of PSF can be applied to create distinct soil microbiomes by growing different plant species in a given soil, which results in different effects on above-ground herbivores that feed on plants that are subsequently grown in these soils^26,30^. To date, there are no studies of PSF-mediated plant responses on herbivorous insects in a dual-attack situation.

In this study, we investigated the induction of the JA and SA-signalling pathways and herbivore interactions in a community context, where plants interact with two herbivore species of different feeding guilds, an aboveground parasitoid, and different belowground soil communities. We used a model system of *Capsicum annuum* var. *maranello* (sweet pepper), the phloem feeding aphid *Myzus persicae* (Sulzer, 1776) (Sternorrhyncha, Aphididae), which is commonly used as a model of SA-inducer, and the cell content feeding thrips *Frankliniella occidentalis* (Pergande, 1895) (Thysanoptera, Tripidae), which is known to induce and be sensitive to JA-regulated defences in Arabidopsis and tomato^31–34^. Both aphids and thrips, are generalist insects and major pests in sweet pepper plants, but also in many other crops worldwide. At the third trophic level, we used the parasitoid *Aphidius colemani* (Dalman, 1820) (Hymenoptera, Braconidae), parasitising the aphid *M. persicae*, in the described plant-herbivore system.

Previous studies have shown that aphid feeding facilitates caterpillar performance, associated with an strong induction of the SA signaling pathway by aphids (although aphids also induce JA signaling) and a suppression of the JA pathway induced by caterpillars^4,6–8^. Based on those studies, and on the fact that parasitism can enhance the induction of JA by caterpillars^11^, we hypothesized that parasitized aphids would induce a stronger JA signaling compared to healthy aphids, interfering with the facilitation of aphids towards thrips. Similarly, we expected that PSF would enhance the JA-plant response to aphids, and therefore resistance to thrips. This hypothesis is based on the fact that PSF are mainly driven by soil microbes^26,35^, and that different soil microbes can prime plants for a stronger JA-responses^3,19,36^. As a consequence, we expected that PSF would also enhance the effects of parasitism on plant signalling (since both above- and belowground factors can enhance a JA-response), with aphids having a more negative effect on thrips than in sterile soil. In order to thest these hypothesis, we addressed two main research questions: (i) Does parasitism of aphids or PSF influence the induction of marker genes of the JA and SA defensive signalling pathways in pepper plants?; (ii) Do these effects of PSF and/or parasitism on plant responses affect the later performance of *F. occidentalis*? By using a model system of agricultural interest we highlight the potential relevance of parasitism at modulating plant responses to aphids and their interaction with thrips.

## Results

### Parasitism alters plant defence responses

**Figure 1:**
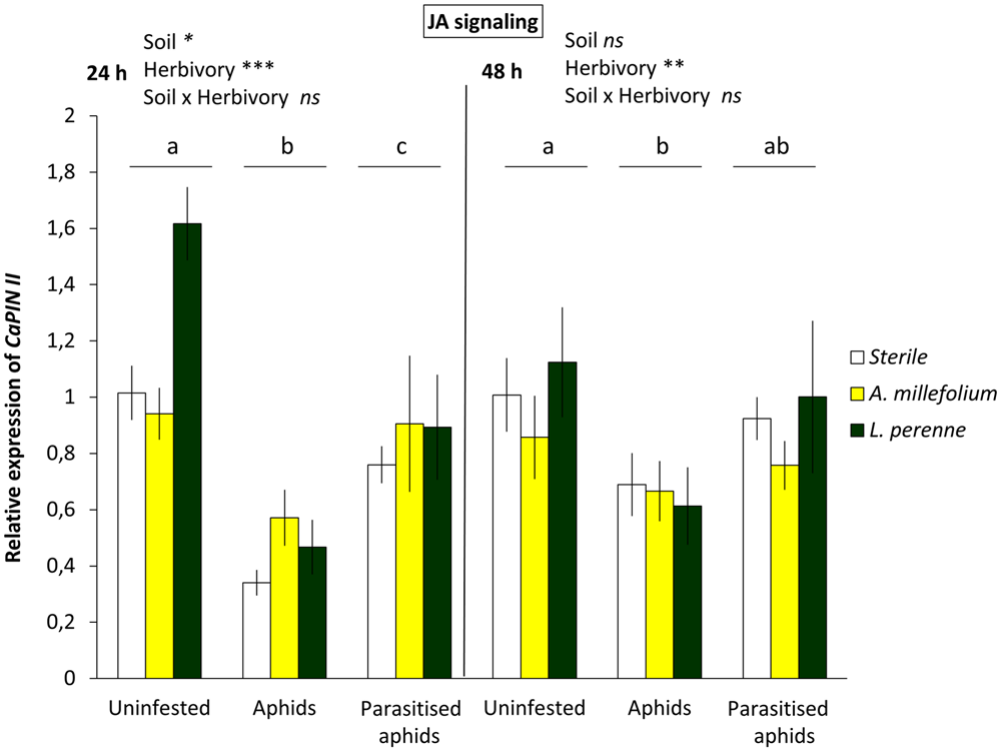
Expression levels of *CaPINII* in *C. annuum* in uninfested, aphid-infested, or parasitised aphids- infested plants, each grown in sterile soil, or inoculated with living soil conditioned by the plants *A. millefolium* or *L. perenne*. Bars represent mean *CaPINII* expression levels normalised as 2^−∆∆Ct^ with standard error bars (n = 4). Bars marked with different letters are significantly different (LSD, P < 0.05), with separate analysis for the two time points (24 and 48 h after aphid infestation).

The transcript levels of the JA-responsive gene *CaPINII* showed a strong down-regulation upon aphid feeding at 24 h and 48 h after infestation (Fig. 1). However, the expression of *CaPINII* was significantly less suppressed by parasitised aphids than by unparasitised ones at 24 h after insect infestation (2-way ANOVA; F = 21.533; *df* = 2, 33; P < 0.001; LSD, P < 0.05; Fig. 1). At 48 h after infestation, healthy aphids still significantly suppressed *CaPINII* expression compared to levels in uninfested plants, while levels in plants with parasitised aphids were similar to both other treatments (2-way ANOVA; F = 3.698; *df* = 2, 34; P = 0.039; LSD, P > 0.05; Fig. 1).

**Figure 2:**
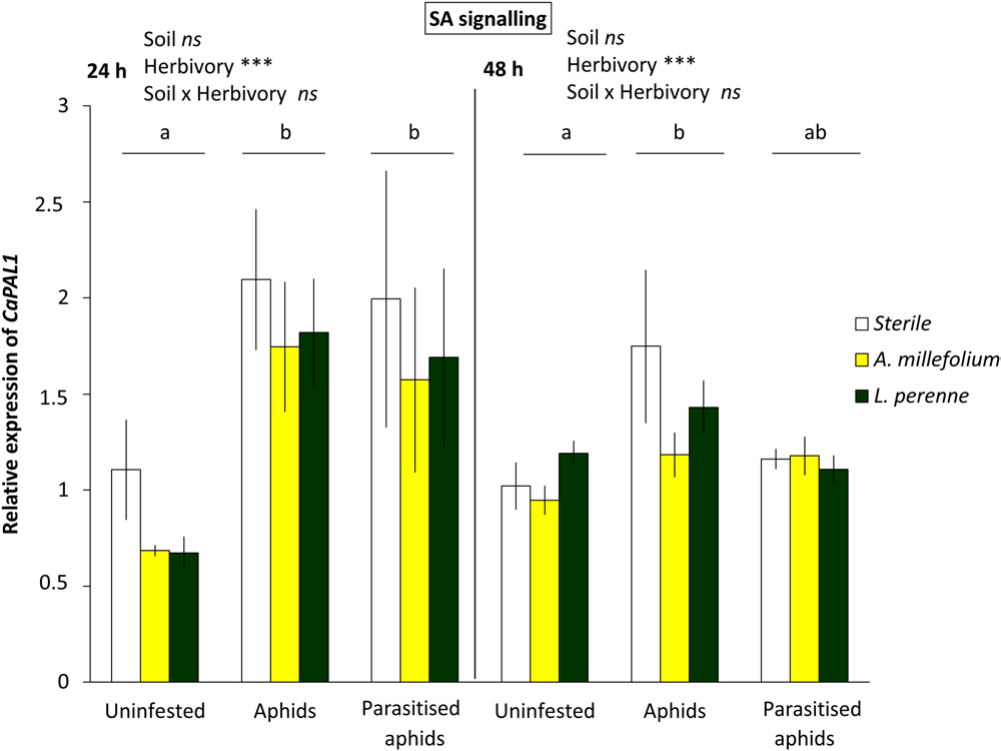
Expression levels of *CaPAL1* in *C. annuum* in uninfested, aphid-infested, or parasitised aphids- infested plants, each grown in sterile soil, or inoculated with living soil conditioned by the plants *A. millefolium* or *L. perenne*. Bars represent mean *CaPAL1* expression levels normalised as 2^−∆∆Ct^ with standard error bars (n = 4). Bars marked with different letters are significantly different (LSD, P < 0.05), with separate analysis for the two time points (24 and 48 h after aphid infestation).

In contrast to *CaPINII*, the transcript levels of *CaPAL1* (SA-biosynthetic gene) were up-regulated upon feeding by both aphids and parasitised aphids, compared with uninfested plants after 24 h of insect infestation (2-way ANOVA; F = 5.684; *df* = 2, 33; P = 0.009; LSD, P < 0.05; Fig. 2). However, at 48 h after insect feeding, only the treatment with unparasitised aphids maintained significantly induced levels of *CaPAL1*, whereas the expression level in plants with parasitised aphids did not differ from uninfested and aphid-infested plants (2-way ANOVA; F = 4.673; *df* = 2, 34; P = 0.018; LSD, P > 0.05; Fig. 2). In contrast to *CaPAL1* and *CaPINII*, the expression of *CaLOX2* (JA-biosynthetic gene) and *CaPR1* (SA-responsive gene) were not up- or downregulated after infestation with (parasitised) aphids (see Supplementary Figs S5 and S6, P > 0.05).

### Plant-soil feedbacks (PSF) do not affect plant defensive hormonal pathways

We investigated the potential effect of three different soil communities on the induction of JA and/or SA defensive genes in sweet pepper plants. The soils selected for the experiment were: soil conditioned with *Achilea millefolium* and *Lolium perenne* and sterile soil as control (see Materials and Methods). No main effect of soil type was found on the expression of *CaLOX2* (JA-biosynthetic gene) (P > 0.05), or SA-marker genes (*CaPAL1* and *CaPR1)*, neither at 24 h nor 48 h after insect infestation (P > 0.05) (Fig. 2, Figs S5 and S6). In contrast, the expression of *CaPINII* (JA-responsive gene) was up-regulated comparing PSF effects in undamaged plants, where soil conditioned by *L. perenne* led to a stronger response than sterile soil at first time point (24 h) (see suppl. statistical results) (2-way ANOVA; F = 3.532; *df* = 2, 33; P = 0.045; Fig. 1). However, the soil effect on the expression of *CaPINII* was no longer observed at 48 h (2-way ANOVA; F = 0.758; *df* = 2, 34; P = 0.479; Fig. 1).

### Aphid herbivory facilitates thrips performance, but not if aphids are parasitised

**Figure 3:**
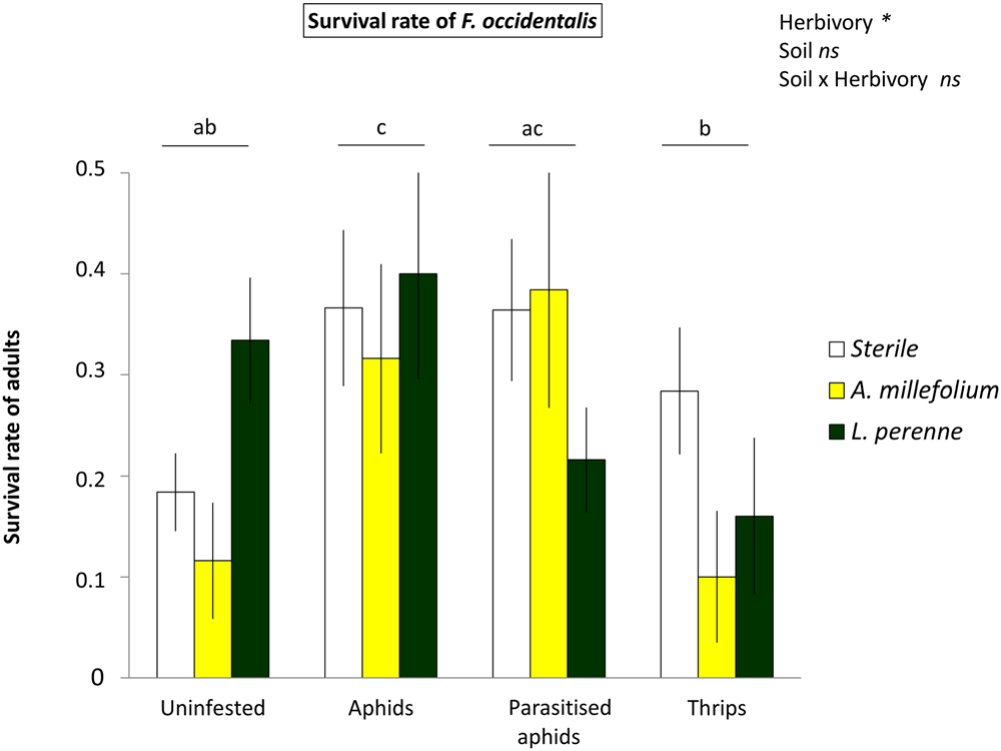
Performance of *F. occidentalis*, number that reach the adult stage (out of 5 initial individuals) on *C. annuum* for four different treatments: (a) uninfested plants, (b) aphid-infested plants, (c) parasitised aphid-infested plants, (d) thrips-infested plants, on three different soil types: (a) sterile soil, (b) *A. millefolium*, (c) *L. perenne*. Bars represent means ± SE (n = 12 replicates). Different letters indicate significant pairwise differences between infestation treatments (P < 0.05).

**Figure 4:**
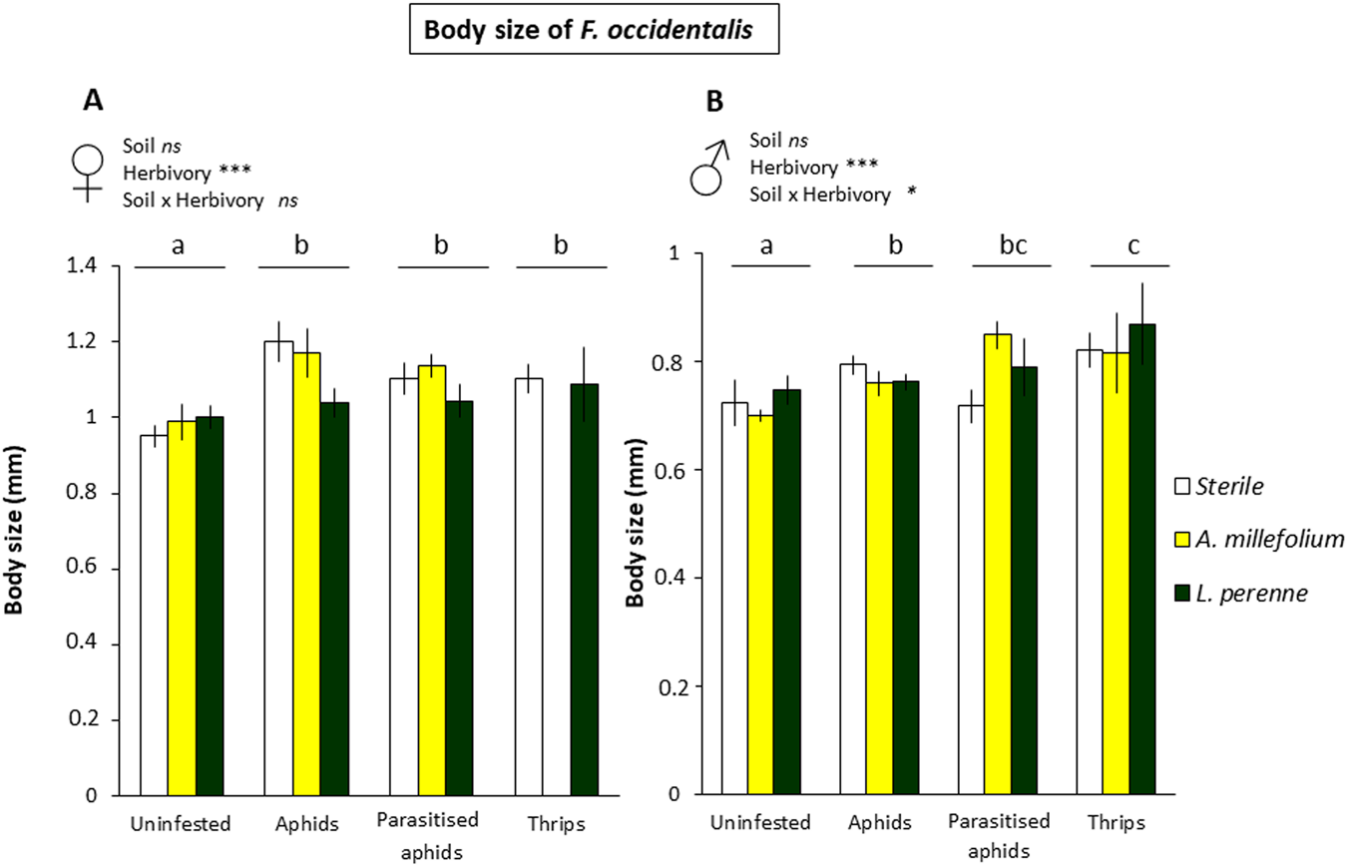
Performance of *F. occidentalis*, length of body size in adult stage for female (**A**) and male (**B**) thrips on *C. annuum* for four different treatments: (a) uninfested plants, (b) aphid infested plants, (c) parasitised aphid infested plants, (d) thrips infested plants, on three different soil types: (a) sterile soil, (b) *A. millefolium*, (c) *L. perenne*. Bars represent means ± SE (n = 12). No surviving females were found in thrips infested plants grown on *A. millefolium* soil. Different letters indicate significant pairwise differences between infestation treatments (P < 0.05).

Thrips survival from two-day-old nymphs until adult stage and length of adult body size were measured as performance parameters of *F. occidentalis*.Thrips were growing on detached leaves from sweet pepper plants previously treated as described above for gene expression analyses. Thrips survival was highest when feeding on leaves from plants that were previously infested by unparasitised aphids (GLM, binomial test; F = 9.491; *df* = 3, 125; P = 0.023; Fig. 3), whereas no differences in survival rates were found for thrips feeding on plants previously infested by parasitised aphids or thrips, compared with uninfested plants. Moreover, a strong effect was observed on the body size of thrips that reached the adult stage. Both females (2-way ANOVA; F = 5.017; *df* = 3, 69; P = 0.004; Fig. 4A) and males (2-way ANOVA; F = 4.470; *df* = 3, 106; P = 0.006; Fig. 4B) were significantly larger when feeding and developing on leaves previously infested by either parasitised or unparasitised aphids, or thrips, compared with uninfested plants. The effect of plant-soil feedbacks was also analysed on thrips performance; but no effect was found on thrips survival nor adult size (P > 0.05). However, a significant interaction was found between soil and herbivory on the body size of thrips males (2-way ANOVA; F = 2.264; *df* = 6, 106; P = 0.044; Fig. 4B), but this interaction was not found in females (2-way ANOVA; F = 1.008; *df* = 5, 69; P = 0.421; Fig. 4A).

## Discussion

This study shows that the plant response to aphid herbivory can be altered by parasitism, but not by legacies left in the soil by previous plants. This pattern also scales-up into the consequences for the survival of a subsequent herbivore feeding on those plants. There is an increasing awareness about how parasitism can alter the plant-defence signalling network and how these changes may have cascading effects at the insect community level. Caterpillar parasitism can increase the feeding damage done by caterpillars, increasing thus the induction of chemical plant defenses with a fitness cost for the plant^14^. Moreover, parasitism of caterpillars can alter both plant gene expression and metabolomics, affecting the foraging decisions made by subsequent herbivores, parasitoids and even hyperparasitoids at the fourth trophic level^11,13^. Our findings demonstrate such differences in plant responses due to parasitism of phloem feeders, which not only have a completely different biology, but also elicit different plant responses. Since parasitoids are indirectly affected by the plant on which their host is feeding^37^ and are mostly immobile inside their hosts, parasitoids may be expected to alter the plant-defence mechanisms for their own benefit. Although it is a not-yet tested hypothesis, a possibility is that parasitoids may alter herbivore induced plant responses to deter other predators that can kill the parasitoids when ingesting their herbivorous prey. This phenomenon of intraguild predation is common between aphid parasitoids and predators, and in a previous study we showed that hoverflies reduce their oviposition on plants colonized by parasitised aphids^38^. In contrast, although not yet known for phloem feeders, hyperparasitoids of caterpillar parasitoids were more attracted to plants infested by parasitised than unparasitised caterpillars^13^. Future studies including multiple members of the different trophic levels may unravel the costs and benefits of the plant responses altered by parasitoids.

Our results show that heterospecific herbivory aboveground by aphids facilitates thrips performance, but not when aphids were parasitised, or when plants were previously infested by conspecific thrips. Several studies with phloem feeders that induce the SA signalling pathway and leaf chewers that induce the JA pathway in a similar way as thrips do, have observed facilitative interactions, mainly associated with cross-talk of these two signalling pathways^5,39–41^. Here, at the plant signalling level, we also observed that aphids suppressed the JA-marker gene *CaPINII*, and associated with this, thrips performance on aphid-infested plants was enhanced. Linked to this result, parasitism mitigated the suppression of *CaPINII*, and also the aphid-triggered facilitation of thrips performance. Further work with pepper plants that have the JA signalling pathway impaired (Sarde *et al*., in prep) could confirm the role of JA pathway in shaping the interactions between multiple attackers in the presence of a member of the third trophic level.

A yet unresolved, question is, which mechanisms in the herbivores are triggering the observed differences between parasitised and unparasitised aphids. In our experiments, we have repeatedly observed that the honeydew covering the plants colonized by parasitised aphids show small white spots that are not present on plants with unparasitised aphids. Aphid honeydew was shown to play a significant role supressing JA accumulation in *Vicia faba* plants^42^. Parasitised aphids may release a higher amount of honeydew than healthy aphids^43^, and therefore a stronger JA suppression by parasitised aphids would be expected. In contrast, our results show a weaker suppression of the JA response in plants colonized by parasitised compared to unparasitised aphids. Thus, we suggest that altered plant responses due to parasitism may be due to changes in honeydew composition rather than quantity. An alternative hypothesis is that the different plant response may be due to changes in aphid saliva, which plays a key role in plant-aphid interactions^44,45^. In caterpillars, parasitism changes the herbivore oral secretions, and this alters plant responses such as the emission of herbivore-induced plant volatiles compounds (HIPVs), which allows parasitoids to discriminate between parasitised and unparasitised hosts, but also allows hyperparasitoids to locate their hosts^11,46,47^. At present, there is no information about differential composition of saliva and honeydew comparing parasitised with healthy aphids.

We did not find evidence of soil treatments altering the relationships between those herbivores nor the plant response (gene expression in JA and SA signalling). In our study, we created different soil microbiomes by applying the concept of plant-soil feedbacks^29,30^, with a methodology that reduces the potential differences in abiotic soil properties (see materials and methods). The selected plants (*L. perenne* and *M. millefolium*) used to condition the soil were selected according to previous results of PSF effects on plant performance and pathogen resistance in chrysanthemum (*Dendranthema X grandiflora*)^48^, and resistance against thrips (Pineda *et al*., in prep.). However, no effect of PSF on induced resistance nor on molecular plant responses were observed in this study with sweet pepper. Plant-soil-insect feedbacks and their underlying mechanisms is a so-far unexplored field, and the effects on insects may vary depending on factors such as plant species, plant communities, or insect feeding guild^26,35^. Further studies on these and other factors affecting plant resistance to herbivores will not only increase our understanding of how plants and insects interact in nature, but also of how to apply the concept of PSF to protect agricultural crops.

The vast majority of studies on plant defences have focused on the responses to the attack of single microbes or herbivores. However, in nature, plants interact with multiple species of attackers and beneficial organisms, calling for increased complexity of the study systems. Using an agriculturally important plant species, our work highlights the role of parasitism at modulating plant defences and heterospecific insect interactions.

## Materials and Methods

### Plants and insects

The study system consisted of Sweet pepper (*Capsicum annuum* var. *maranello)*, an organically certified cultivar commonly used in organic greenhouse crops, the generalist phloem feeding aphid *M. persicae*, the cell-content feeding thrips *F. occidentalis* and *A. colemani* as the parasitoid of *M. persicae*. For the soil conditioning, we selected the forb *Achilea millefolium* L. (Asteraceae) and the grass *Lolium perenne* L. (Poaceae), species that in a previous study on chrysanthemum led to a thrips-suppressive plant-soil feedback effect (Pineda *et al*., in prep.).

Insects were reared at NIOO-KNAW, Wageningen, The Netherlands. *Myzus persicae* was reared on *C. annuum* plants for multiple generations, *A. colemani* was provided by Koppert Biological System and *F. occidentalis* was reared on fresh green beans *Phaseolus vulgaris* L. (Fabaceae), in climate chambers at 22 °C ± 2 °C, 40% relative humidity (RH) and a 16 h light and 8 h dark photo regime.

### Soil preparation

To create distinct soil microbiomes, living soil collected from a grassland from the national park The Hoge Veluwe (The Netherlands) was conditioned by the wild plants *A. millefolium* and *L. perenne*, as described in Kos *et al*.^27^. One seedling per pot was transplanted into this soil (12 replicates per each wild plant), and pots were randomly located inside a greenhouse (21/16 °C day/night, 16 h photoperiod). Plants were watered three times per week. Natural daylight was supplemented by 400 W metal halide lamps (225 µmol m ^−2^ s ^−1^ PAR). After 8 weeks of conditioning phase, soil inocula were collected, keeping each soil replicate separately.

Then the soil inocula were mixed with sterilized bulk soil (10% soil inocula: 90% sterilized soil) and for control soil only sterilized bulk soil was used. To obtain sterilized bulk soil, the same field soil was sterilized by gamma irradiation (>25 KGray: Isotron, Ede, The Netherlands). Pots (13 × 13 × 13 cm) were filled with 1 kg of mixed soil in total. The mixing of inocula with sterilized soil reduces differences in the abiotic characteristics of the soil, while the biotic component can recolonize the sterile soil^25^. To ensure that at least one seedling of sweet pepper survived, we used two seeds per pot. The seeds were surface-sterilized (1 min in 0.1% sodium chloride solution and rinsed with water) and germinated directly in the pots. After two weeks, only one seedling of sweet pepper was kept per pot. Plants were grown in a greenhouse under the same conditions as described above. Plants were watered three times per week, supplied with nutrient solution (Hoagland). In total, there were 288 pots (4 herbivore treatments × 3 soil treatments × 12 replicates × 2 time points).

### Insect infestation and harvesting

Five weeks after germination, plants were randomly allocated to one of the following treatments (i) uninfested, (ii) aphid-infested, (iii) parasitised aphid-infested and (iv) thrips-infested. All treatments were replicated 12 times per soil treatment and two groups were labelled as 24 h and 48 h. All the plants were individually covered with gauze cages to prevent the escape of insects, and uninfested plants were covered as well to standardise conditions. For the treatment of aphid-infested plants, thirty three-day-old nymphs of *M. persicae* were placed on the second expanded leaf from each plant. For the treatment of parasitised aphid-infested plants, a colony of *A. colemani* was allowed to parasitise approximately 1500 two-day-old nymphs of *M. persicae* during 24 h. Then, thirty of these three-day-old newly parasitised aphids were placed on each plant (99% of parasitism rate was confirmed after material collection). Parasitized aphids become mummies and stop feeding at the fourth instar or adult stage^49^, which occurs around 10 days after parasitoid oviposition^50^. Thus during this experiment parasitized aphids were feeding, since they were parasitized only 4 and 5 days before. For the thrips-infested plants treatment, ten three-day-old nymphs of *F. occidentalis* were enclosed in a clip cage on the plant, to ensure that thrips did not escape through the gauze. Results of gene expression from thrips-infested plants were analysed separately (see suppl. Materials) because of the use of clip cages in that single treatment, which can damage superficially the leaf and therefore interfere in the gene expression results compared with the control plants.

At 24 and 48 h after insect infestation, one leaf disc of 1 cm diameter was harvested per plant, using the second expanded leaf. In the plants with aphid infestation, we carefully removed their exuviae with a fine paintbrush prior to the collection. Uninfested plants were harvested similarly to the infested plants. Four separate biological replicates were arranged per treatment, and each replicate consisted of a pool of three leaf discs from three individual plants (randomly pooled). The collected material was labelled as 24 h or 48 h harvested and immediately frozen in liquid nitrogen and stored at −80 °C for RNA isolation.

### RNA extraction, cDNA synthesis and quantitative RT-qPCR reaction

Total RNA extraction and purification was done following the protocol of Isolate II RNA Plant Kit (Bioline, London, United Kongdom). After purification, the RNA concentration and purity were measured using a NanoDrop ND-100 (NanoDrop Technologies, Wilmington, DE, USA) spectrophotometer (all samples with OD_260/280_ = 1.9–2.1), and RNA integrity was confirmed by gel electrophoresis. Isolated RNA was converted into cDNA using the iScript cDNA synthesis Kit (Biorad, Hercules, CA, USA), and diluted 1:20 with RNase free water.

Quantitative RT-qPCR analysis was used to evaluate the expression profiles of two genes involved in the JA-signalling pathway (*CaLOX2* and *CaPINII*) and two genes involved in the SA-pathway (*CaPAL1* and *CaPR1*), in which are appropriated markers in sweet pepper plants and common markers of the SA and JA pathways in other systems (Sarde *et al*. in prep) (see Supplementary information Table S1 for primer sequences). In addition, the expression of the reference genes *CaUEP* and *CaACTIN* was assessed for normalization (see further methods in Supplementary information).

### Thrips performance experiment

Nymphs of *F. occidentalis* were allowed to develop until the adult stage while feeding on sweet pepper plants with previous herbivory (either from thrips, aphids or parasitised aphids), and growing on the different soils. From the same plants that were infested for 48 h and a sample was taken for molecular analyses, the fourth entire leaf was used for the performance bioassay (see supplementary methods: Thrips performance). The leaf petiole from each plant was inserted in 2 ml 1.5% plant agar in a 90 mm Petri dish, to maintain leaf freshness. Using a fine paintbrush, five two-day-old nymphs of *F. occidentalis* were transferred to each Petri dish. In total, there were 144 plates (4 herbivore treatments × 3 soil treatments × 12 replicates) and 720 individuals of thrips (5 nymphs × 144 samples). The thrips were monitored daily, starting 4 days later and until they became adults (±7 days monitoring). Survival and length of adult body-size, measured from head until the last part of the abdomen by a digital microscope (SZX12 Olympus; Tokyo, Japan), was recorded (due to differences between males and females, body size measurements were analyzed separately for each sex). The bioassay was performed in a growth chamber at 22 °C, 40% relative humidity (RH) and a 16 h light and 8 h dark photo regime.

### Statistical analyses

After confirmation of the assumptions of normality and homogeneity of variances, differences in gene expression levels between previous herbivory attack and plant-soil feedbacks were analysed using two-way ANOVA’s, where herbivory and soils were set as fixed factors. Analysis were done separately for the different time points. The same two-way ANOVA models were applied to analyse the differences in thrips body size, with separate analysis for males and females, after averaging measurements from individuals from each replicate (Petri dish). To analyse whether induction by PSF and/or sequential herbivory affected thrips survival we used generalised linear models (GLM), with logit link function and binomial distribution, and the dispersion parameter estimated to correct for over-dispersion. All pairwise comparisons were done with the post-hoc protected LSD test (SPSS 15.0.; SPSS Inc., Chicago, II, USA).

## Electronic supplementary material

Supplementary information

## References

[1] Walling LL (2000). The myriad plant responses to herbivores. J. Plant Growth Regul..

[2] De Vos M (2005). Signal signature and transcriptome changes of *Arabidopsis* during pathogen and insect attack. Mol. Plant Mic. Int..

[3] Pieterse CMJ (2012). S. C. M. Hormonal modulation of plant immunity. Annu. Rev. Cell Dev. Biol..

[4] Rodriguez-Saona CR, Musser RO, Vogel H, Hum-Musser SM, Thaler JS (2010). Molecular, biochemical, and organismal analyses of tomato plants simultaneously attacked by herbivores from two feeding guilds. J. Chem. Ecol..

[5] Soler R (2012). Plant-mediated facilitation between a leaf-feeding and a phloem-feeding insect in a brassicaceous plant: from insect performance to gene transcription. Funct. Ecol..

[6] Ali JG, Agrawal AA (2014). Asymmetry of plant-mediated interactions between specialist aphids and caterpillars on two milkweeds. Funct. Ecol..

[7] Pineda A, Soler R, Pastor V, Li Y, Dicke M (2017). Plant-mediated species networks: the modulating role of herbivore density. Ecol. Entomol..

[8] Ponzio C, Gols R, Weldegergis BT, Dicke M (2014). Caterpillar-induced plant volatiles remain a reliable signal for foraging wasps during dual attack with a plant pathogen or non-host insect herbivore. Plant Cell Environ..

[9] Ode PJ (2006). Plant chemistry and natural enemy fitness: effects on herbivore and natural enemy interactions. Annu. Rev. Entomol..

[10] Gols R (2014). Direct and indirect chemical defences against insects in a multitrophic framework. Plant Cell Environ..

[11] Poelman EH (2011). Parasitoid-specific induction of plant responses to parasitized herbivores affects colonization by subsequent herbivores. Proc. Natl. Acad. Sci..

[12] Zhu F, Poelman EH, Dicke M (2014). Insect herbivore-associated organisms affect plant responses to herbivory. New. Phyt..

[13] Zhu F (2015). Parasitism overrides herbivore identity allowing hyperparasitoids to locate their parasitoid host using herbivore-induced plant volatiles. Mol. Ecol..

[14] Ode PJ, Harvey JA, Reichelt M, Gershenzon J, Gols R (2016). Differential induction of plant chemical defenses by parasitized and unparasitized herbivores: consequences for reciprocal, multitrophic interactions. Oikos.

[15] Poelman EH (2011). Indirect plant-mediated interactions among parasitoid larvae. Ecol. Lett..

[16] Yang J, Kloepper JW, Ryu CM (2009). Rhizosphere bacteria help plants tolerate abiotic stress. Trends Plant Sci..

[17] Pineda A, Zheng SJ, van Loon JJA, Pieterse CMJ, Dicke M (2010). Helping plants to deal with insects: the role of beneficial soil-borne microbes. Trends Plant Sci..

[18] Jung SC, Martinez-Medina A, Lopez-Raez JA, Pozo MJ (2012). Mycorrhiza-induced resistance and priming of plant defenses. J. Chem. Ecol..

[19] Pieterse CMJ (2014). Induced systemic resistance by beneficial microbes. Annu. Rev. Phyto..

[20] Pangesti N (2016). Jasmonic acid and ethylene signaling pathways regulate glucosinolate levels in plants during rhizobacteria-induced systemic resistance against a leaf-chewing herbivore. J. Chem. Ecol..

[21] Bezemer TM (2005). Soil community composition drives aboveground plant-herbivore-parasitoid interactions. Ecol. Lett..

[22] Erb M (2009). Signal signature of aboveground-induced resistance upon belowground herbivory in maize. Plant J..

[23] Soler R (2012). Root herbivore effects on aboveground multitrophic interactions: patterns, processes and mechanisms. J. Chem. Ecol..

[24] Bezemer TM, Van Dam NM (2005). Linking aboveground and belowground interactions via induced plant defenses. Trends Ecol. Evol..

[25] Kostenko O, van de Voorde TFJ, Mulder PPJ, van der Putten WH, Bezemer TM (2012). Legacy effects of aboveground-belowground interactions. Ecol. Lett..

[26] Bezemer TM (2013). Above- and below-ground herbivory effects on below-ground plant-fungus interactions and plant-soil feedback responses. J. Ecol..

[27] Kos M, Tuijl MAB, de Roo J, Mulder PPJ, Bezemer TM (2015). Species-specific plant-soil feedback effects on above-ground plant-insect interactions. J. Ecol..

[28] Bartelt-Ryser J, Joshi J, Schmid B, Brandl H, Balser T (2005). Soil feedbacks of plant diversity on soil microbial communities and subsequent plant growth. Perspect. Plant Ecol..

[29] Ehrenfeld JG, Ravit B, Elgersma K (2005). Feedback in the plant-soil system. Annu. Rev. Environ. Resour..

[30] Van der Putten WH (2013). Plant-soil feedbacks: the past, the present and future challenges. J. Ecol..

[31] Abe H (2008). Function of jasmonate in response and tolerance of Arabidopsis to thrip feeding. Plant Cell Physiol..

[32] Abe H (2009). Jasmonate-dependent plant defense restricts thrips performance and preference. BMC Plant Biol..

[33] Kawazu K (2012). Different expression profiles of jasmonic acid and salicylic acid inducible genes in the tomato plant against herbivores with varius feeding modes. Arthropod-Plant Interact..

[34] Bravo-Escobar R, Klinkhamer PGL, Leiss KA (2017). Induction of jasmonic acid-associated defenses by thrips alters host suitability for conspecifics and correlates with increased trichome densities in tomato. Plant Cell Physiol..

[35] Heinen Robin, van der Sluijs Martijn, Biere Arjen, Harvey Jeffrey A., Bezemer T. Martijn (2017). Plant community composition but not plant traits determine the outcome of soil legacy effects on plants and insects. Journal of Ecology.

[36] Pineda A, Kaplan I, Bezemer TM (2017). Steering soil microbiomes to suppress aboveground insect pests. Trends Plant Sci..

[37] Bottrell DG, Barbosa P, Gould F (1998). Manipulating natural enemies by plant variety selection and modification: a realistic strategy?. Annu. Rev. Entomol..

[38] Pineda A, Morales I, Marcos-García MA, Fereres A (2007). Oviposition avoidance of parasitized aphid colonies by the syrphid predator *Episyrphus balteatus* mediated by different cues. Biol. Control.

[39] Stout MJ, Workman KV, Bostock RM, Duffey SS (1997). Specificity of induced resistance in the tomato. Lycopersicon esculentum. Oecologia.

[40] Rodriguez-Saona C, Chalmers JA, Raj S, Thaler JS (2005). Induced plant responses to multiple damagers: differential effects on an herbivore and its parasitoid. Oecologia.

[41] Li Y, Dicke M, Harvey JA, Gols R (2014). Intra-specific variation in wild B*rassica oleracea* for aphid-induced plant responses and consequences for caterpillar-parasitoid interactions. Oecologia.

[42] Schwartzberg EG, Tumlinson JH (2014). Aphid honeydew alters plant defence responses. Funct. Ecol..

[43] Völkl W (1992). Aphids or Their Parasitoids: Who Actually Benefits from Ant-Attendance?. J. Anim. Ecol..

[44] Mutti NS (2008). A protein from the salivary glands of the pea aphid, *Acyrthosiphon pisum*, is essential in feeding on a host plant. Proc. Natl. Acad. Sci.USA.

[45] De Vos M, Jander G (2009). *Myzus persicae* (green peach aphid) salivary components induce defence responses in *Arabidopsis thaliana*. Plant Cell Environ..

[46] Fatouros NE, Van Loon JJA, Hordijk KA, Smid HM, Dicke M (2005). Herbivore-induced plant volatiles mediate in-flight host discrimination by parasitoids. J. Chem. Ecol..

[47] Poelman EH (2012). Hyperparasitoids use herbivore-induced plant volatiles to locate their parasitoid host. PLoS Biol..

[48] Ma H-K, Pineda A, van der Wurff AWG, Raaijmakers C, Bezemer TM (2017). Plant–soil feedback effects on growth, defense and susceptibility to a soil-borne disease in a cut flower crop: species and functional group effects. Front. Plant Sci..

[49] Perdikis DCH, Lykouressis DP, Garantonakis NG, Iatrou SA (2004). Instar preference and parasitization of *Aphis gossypii* and *Myzus persicae* (Hemiptera:Aphididae) by the parasitoid *Aphidius colemani* (Hymenoptera: Aphididae). Eur. J. Entomol..

[50] Zamani AA, Talebi A, Fathipour Y, Beniamen V (2007). Effect of temperature on life history of *Aphidius colemani* and *Aphidius matricariae* (Hymenoptera:Braconidae), two parasitoids of *Aphis gossypii* and *Myzus persicae* (Homoptera:Aphididae). Environ. Entomol..

