# Supplementary material for: Modulation of plant-mediated interactions between herbivores of different feeding guilds: Effects of parasitism and belowground interactions
Source: Sci Rep. 2018 Sep 26;8:14424. doi: 10.1038/s41598-018-32131-9 (PMC6158277; doi:10.1038/s41598-018-32131-9)
Supplement: Supplementary file 1 — Supplementary information [file 41598_2018_32131_MOESM1_ESM.docx]

## Supplementary Information

**Modulation of plant-mediated interactions between herbivores of different feeding guilds: Effects of parasitism and belowground interactions**

Teresa Vaello^1^; Sandeep J Sarde^2^; Mª Ángeles Marcos-García^1^; Jetske G. de Boer^3^ ; Ana Pineda^3^

1. Unidad Asociada IPAB (UA-CSIC), Instituto Universitario de Investigación CIBIO, University of Alicante. Ctra. San Vicente del Raspeig s/n, E-03690. Alicante, Spain

*Corresponding author.

Teresa Vaello

Tel: + 34 965 90 9607

Fax: + 34 965 90 3780

E-mail: [mtvl1@alu.ua.es](mailto:mtvl1@alu.ua.es)

The following Supporting Information is available for this article:

**Supplementary methods**

**Thrips performance on 2^nd^ and 4^th^ leaf**

A previous experiment was done to observe the performance of *F. occidenatalis* on different sweet pepper leafs. We use the same conditioned soil that in the main manuscript (*A.millefolim*, *L.perenne* and Sterile soil). Nymphs of *F. occidentalis* were allowed to develop until adult stage while feeding on leaves from previous uninfested conditioned-soil sweet pepper plants. Second and fourth expanded leaf, were used for the bioassay. The leaf petiole from each plant was inserted in 2 ml 1.5% plant agar in a 90 mm petri dish, to maintain leaf freshness. Using a fine paintbrush, five two-day-old nymphs of *F. occidentalis* were transferred to each petri dish. The thrips were then monitored daily starting 4 days later and until they became adults (± 7 days monitoring). Survival and length of adult body-size was recorded. In total, there were 90 replicates (3 soil treatments x 12 soil replicates x 2 leaf stage) and 450 individuals of thrips observed (5 nymphs x 90 replicates). The bioassay was performed in a growth chamber at 22 °C, 40% relative humidity (RH) and a 16 h light and 8 h dark photo regime.


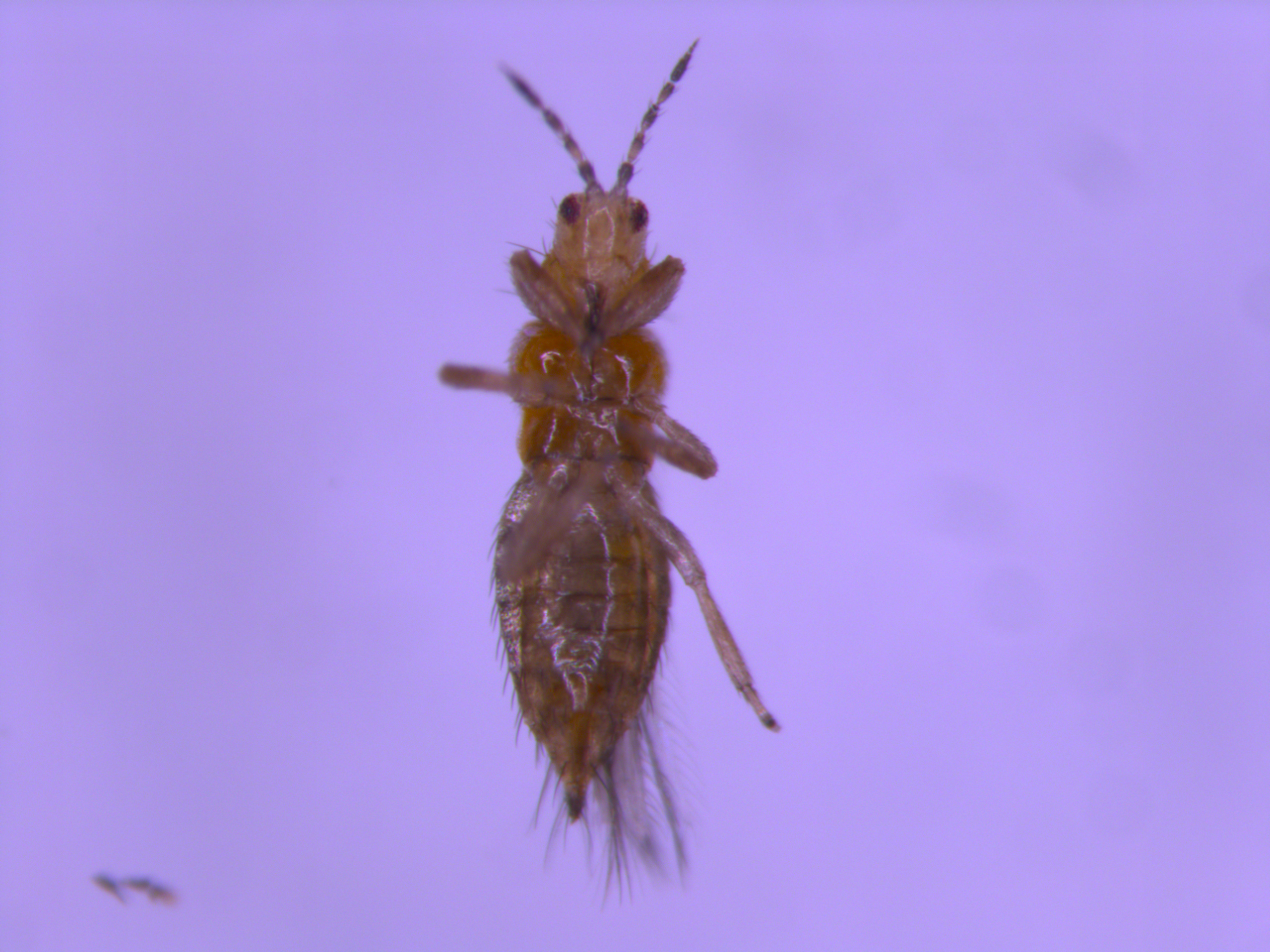


**Measurement of thrips body size length**

**Supplementary results**

**Fig. S5.**

**
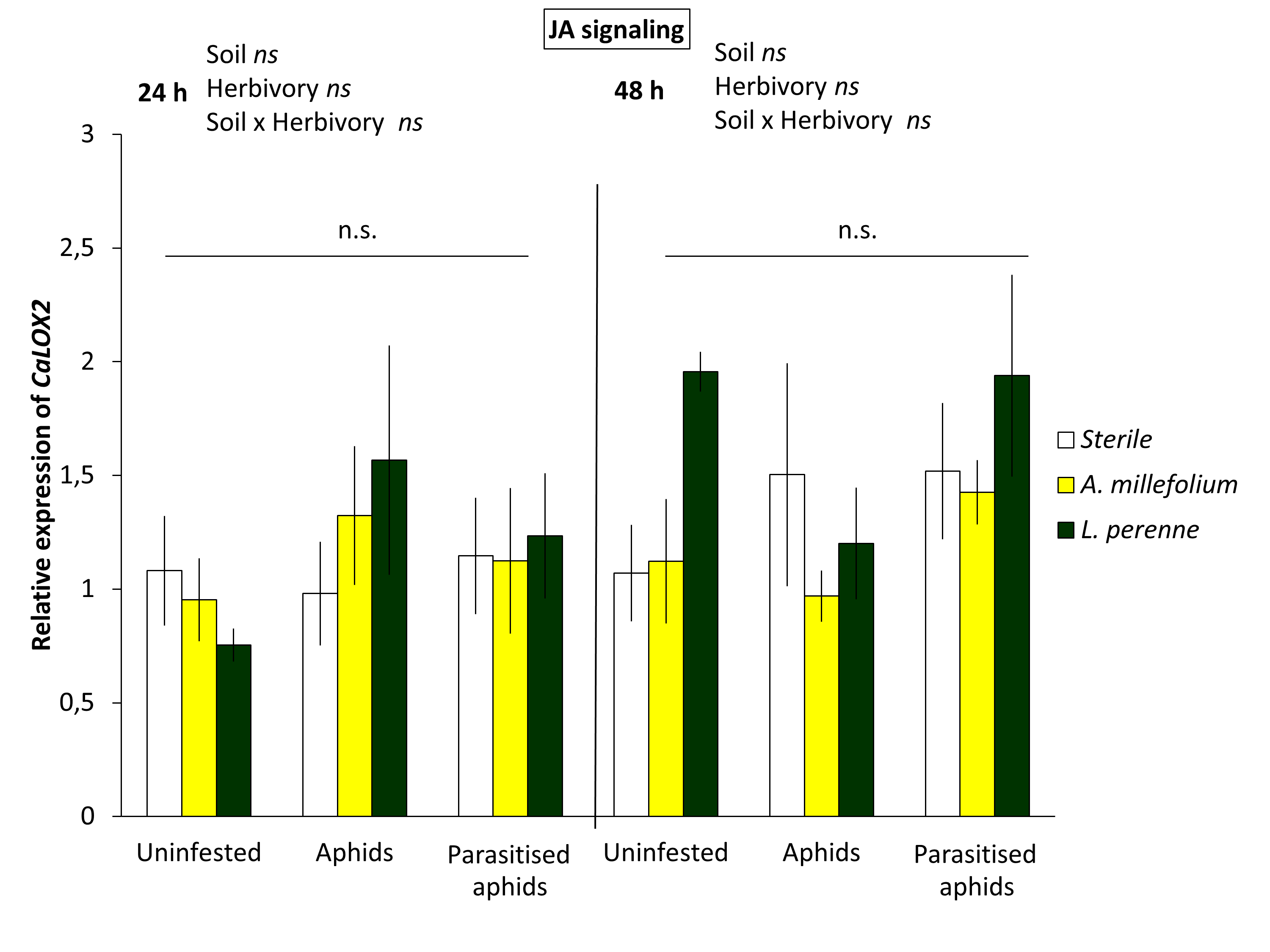
**

Fig S5. Expression levels of *CaLOX2*  in *C. annuum* in a) uninfested plants, b) aphids infested plants, c) parasitised aphids infested plants, in three different soil treatments a) sterile soil, b) *A. millefolium*, c) *L. perenne*; for 24 and 48H. Bars represent mean *CaLOX2* expression levels normalised (by geometrically averaging the Ct values from the genes *CaUEP* and *CaACTIN*, and referred to the control uninfested treatment) as 2^-∆∆Ct^ with standard error bars (n = 4). Bars marked with ns are non significantly different (P > 0.05).

**Fig. S6.**

**
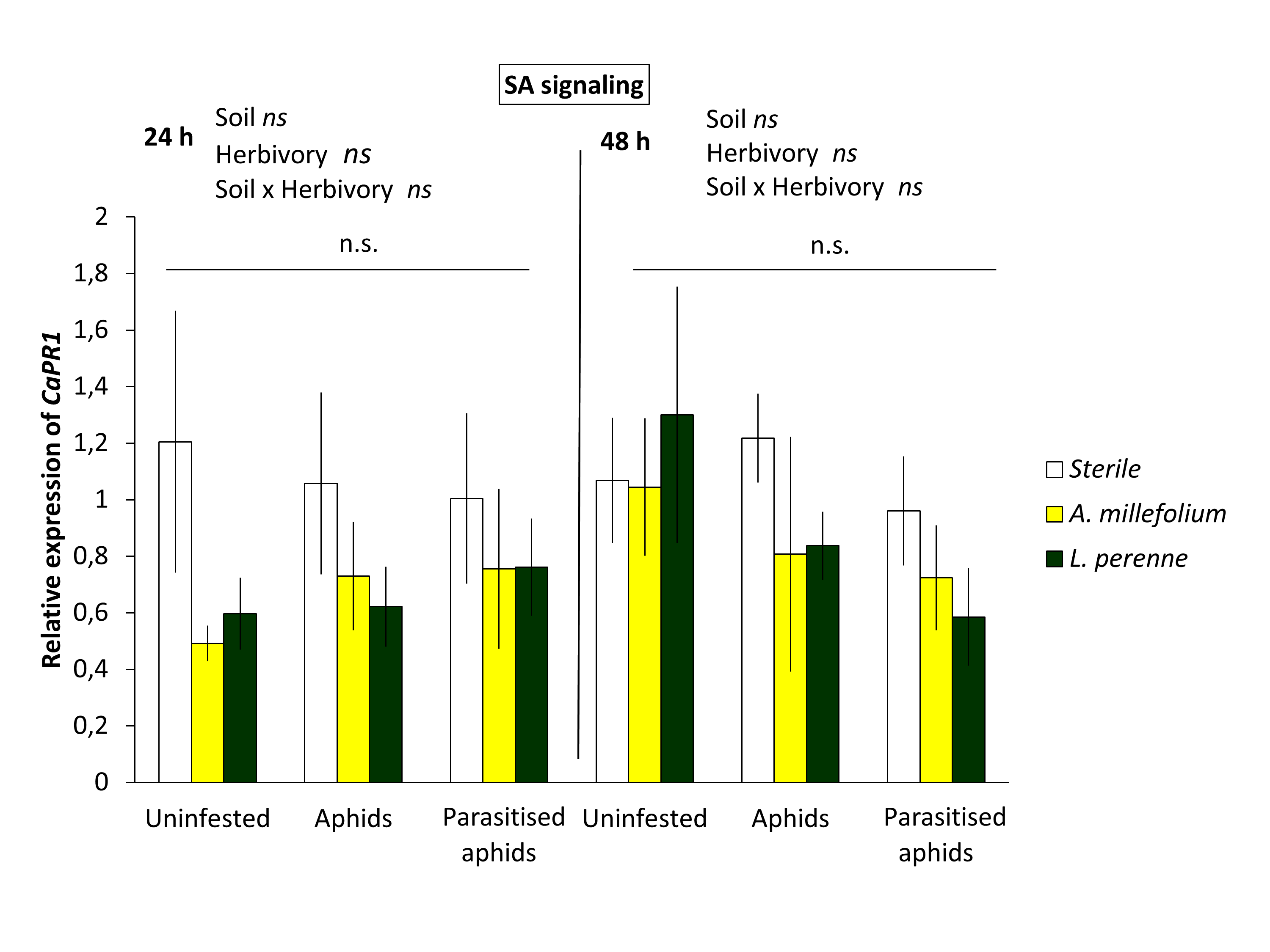
**

Fig S6. Expression levels of *CaPR1* in *C. annuum* in a) uninfested plants, b) aphids infested plants, c) parasitised aphids infested plants, in three different soil treatments a) sterile soil, b) *A. millefolium,* c) *L. perenne;* for 24 and 48H. Bars represent mean *CaPR1* expression levels normalised (by geometrically averaging the Ct values from the genes *CaUEP* and *CaACTIN*, and referred to the control uninfested treatment) as 2^-∆∆Ct^ with standard error bars (n = 4). Bars marked with n.s are non significantly different (P > 0.05).

**Fig. S7. PSF did not affect the induction of marker genes upon thrips attack**


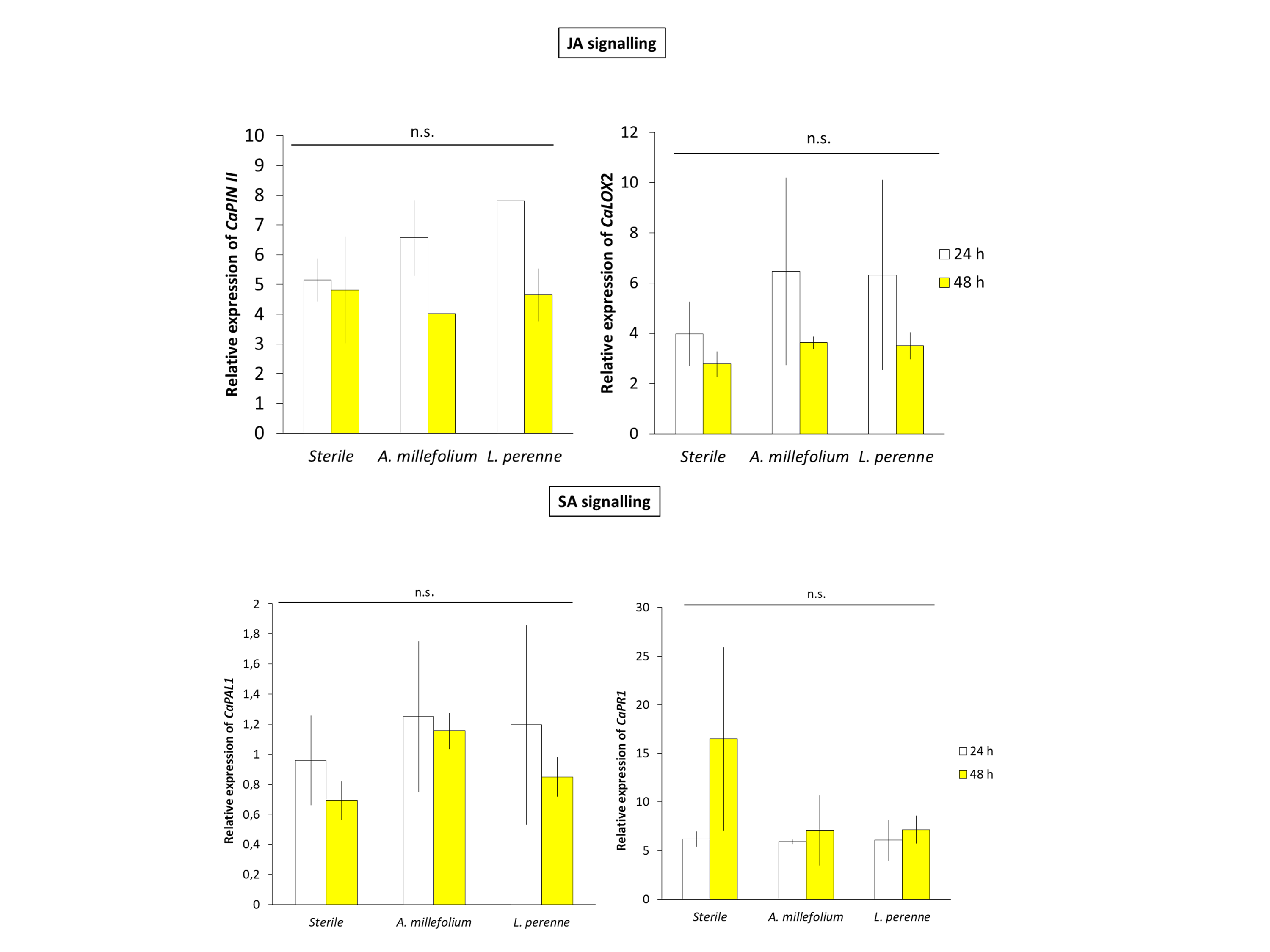
We analysed separately the results of plant defence response with thrips because of the use of clipcages, which can damage superficially the leaf and therefore interfere in the gene expression results compared with the control plants. PSF not had effects on the regulation in any of the responsible genes of SA or JA signalling pathway (P>0.05).

**Fig. S8. PSF effect on survival rate of F. occidentalis**

To evaluate whether PSF and/or the effect of younger or older leaves may had in *F. occidentalis* performance, the survival of thrips from nymphs to adults stage and their final body size on sterile soil, *A.millefolium* and *L.perenne* soil, was tested. The results show how *F. occidentalis* had a lower survival rate when fed on the second leaf (young leaf) from sweet pepper plant compared with individuals which were fed on fourth leaf (old leaf) (GLM; binomial test; F: 14.868; *df*: 1; P <0.001). However, no effects of PSF were found on the survival rate, (GLM; binomial test; F: 1.442; *df*:2 ; P =>0.05)

We did not found body size effects neither on males nor females (P =>0.05). Thus, we decided to use the fourth leaf,for the main experiment, due to the higher survival rate results.


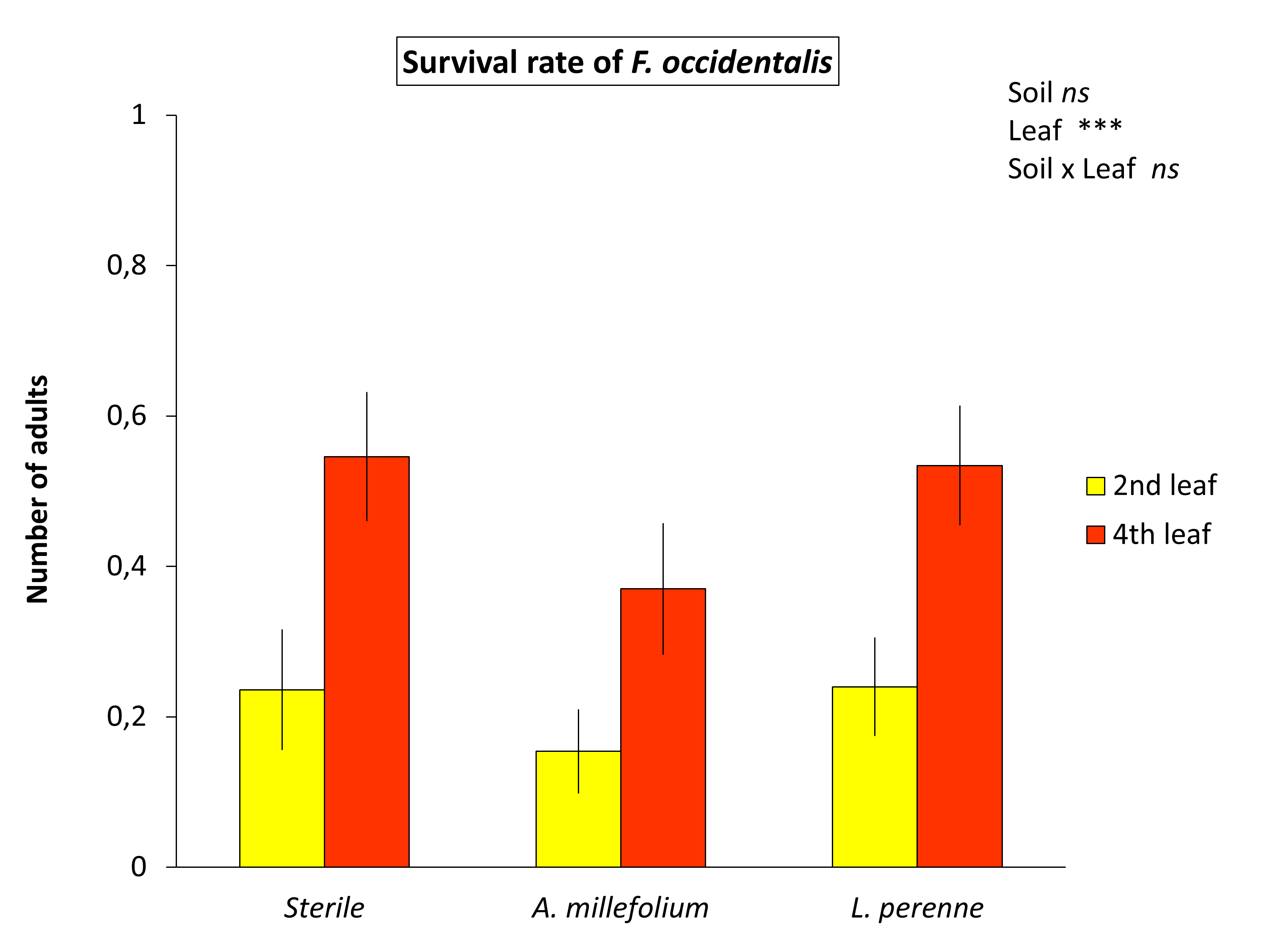


**Supplementary qPCR reaction information**

All qPCR reactions were performed in a Biorad CFX Thermocycler (Biorad, Hercules, CA, USA). The amplification reactions were performed in 20 µl final volume containing 10 µl SensiFAST SYBR No-ROX mix (Bioline, London, United Kingdom), 0.8 µl forward primer and reverse primer [concentration see Supporting Information TableS1], 3.4 µl RNase free water and 5 µl cDNA. The following PCR protocol was used for all PCR analyses with exception of reactions containing the primer for *CaLOX2*: 95 ºC for 2 min, followed by 40 cycles of 95 ºC for 5 s and 30 s at 60 ºC. For reaction mixes containing *CaLOX2* primers the same protocol was used with 95 ºC for 2 min, followed by 40 cycles of 95 ºC for 5 s and 30 s at 62 ºC. All reactions were performed in duplicate and average values were used in the analyses. Reactions efficiencies and Ct values were calculated using the LinRegPCR software (version 2014.7). The most stable reference was calculated from the two reference genes by repeated pair-wise correlation analysis using the Excel-based tool BestKeeper (Pfaffl et al. 2004). Duplicate samples were averaged and expression was normalized by substracting the BestKeeper reference. The gene expression was further normalized against the control treatment of the respective pool of treatment replicate using the comparative Ct-method (also known as 2^-∆∆Ct^ method) (Livak and Schmittgen 2001).

**Table S1: Primer sequences for RT-qPCR**

Sequences of all primers used in RT-qPCR analysis

| Gene | Forward primer (5’-3’) | Reverse primer (5’-3’) | Concentration (μM) | |
| --- | --- | --- | --- | --- |
| *CaUEP* | CCGACTACAACATCCAGAAG | CACACTCAGCATTAGGACAC | 7.5 |  |
| *CaACTIN* | CCCAGATTATGTTTGAGACC | GCAAAGCATAACCCTCATAG | 7.5 |  |
| *CaPAL1* | GGAAATGGCTGCTGAATCAT | GCTCCACTTTAACCCCACAA | 7.5 |  |
| *CaLOX2* | GCCATTTCTGGATCGGATTA | GCATCAACAGGTGGTGTGAC | 7.5 |  |
| *CaPR1* | CCTTACGGGGAAAACCTAGC | ACCCTAGCACAACCAAGACG | 7.5 |  |
| *CaPIN II* | TGGCTGTTCCCAAAGAAGTT | GGTCAGACTCTCCTTCACAA | 7.5 |  |
